# Tyrosinemia type 1 and ADHD like symptoms similarity or comorbidity about a case

**DOI:** 10.1192/j.eurpsy.2023.1081

**Published:** 2023-07-19

**Authors:** H. Belhadga, Z. Elmaataoui, H. Kisra

**Affiliations:** Hôpital psychiatrique universitaire arrazi de sale, Hôpital psychiatrique universitaire arrazi de Sale, Sale, Morocco

## Abstract

**Introduction:**

Many metabolic diseases influence brain function and are associated with psychiatric symptoms and neuropsychiatric disorders (including autism-spectrum disorders, ADHD and psychotic disorders). Attention-deficit-/hyperactivity disorder (ADHD) is among the most common neurodevelopmental disorders in children, with a worldwide prevalence of about 5% in childhood. Tyrosinemia is caused by a genetic mutation in the fumarylacetoacetase gene that leads to a deficiency in the encoded enzyme, which catalyzes the cleavage of tyrosine metabolites to acetoacetic acid and fumaric acid. In recent studies of children with tyrosinemia type 1, a strong correlation was observed between symptoms of ADHD and blood levels of tyrosine, supporting a direct role of this amino acid in the pathogenesis.

**Objectives:**

we report this case of tyrosinemia type 1 associated to ADHD symptoms to contribute in literature to provide more insights into possible shared pathophysiological mechanisms and how these affect their treatment.

**Methods:**

We report the case of an 8-year-old child, followed since the age of 3 months for a tyrosinemia type 1 who presented symptoms of ADHD.

**Results:**

scales and questionnaires were used to detect ADHD symptoms, the **SNAP IV - Swanson, Nolan and Pelham Teacher and Parent Rating Scale** was used with the mother, the items concerning inattention (items 1 to 10) and Hyperactivity-Impulsivity (items 11 to 20) were revealing; The **Conners Evaluation Questionnaire** was delivered, confirming the same result, a **neuropsychological evaluation of the child with IQ evaluation by WISC-IV - Wechsler Intelligence Scale** for Children and Adolescents revealed limited intellectual performance with an IQ of 65.

**Conclusions:**

NMDs, such as HT-1, constitute a large group of conditions that are often containable with early clinical intervention, but still present lifelong difficulties and high societal costs. many studies suggest that there may be similar biological mechanisms behind the cognitive difficulties seen in ADHD and HT-1. In clinical settings, the impaired dopamine synthesis due to substrate inhibition in treated HT-1 may be compensated for by standard ADHD medication, such as methylphenidate or amphetamine. Similarly, the reduced serotonin synthesis may be counteracted by tryptophan supplementation.

**Disclosure of Interest:**

None Declared

